# MIR9-3HG/let-7c-5p/THBS1 axis promotes hepatocellular carcinoma progression

**DOI:** 10.1186/s41065-026-00694-7

**Published:** 2026-06-04

**Authors:** Jinmin Su, Jiaoling Gu, Zhizhong Lu

**Affiliations:** 1 Department of Clinical Laboratory, The 966th Hospital of The PLA Joint Logistic Support Forse, Liaoning, 118000 China; 2 Department of Clinical Laboratory, Jiaozuo People’s Hospital, No, 267, Jiefang Middle Road, Jiaozuo, 454002 China

**Keywords:** Hepatocellular carcinoma, MIR9-3HG, hsa-let-7c-5p, THBS1

## Abstract

**Background:**

Long non-coding RNAs (lncRNA) are key regulators in hepatocellular carcinoma (HCC). However, the role of MIR9-3HG in HCC remained unclear.

**Objective:**

This study investigated the expression, clinical significance, and molecular mechanism of MIR9-3HG in HCC.

**Methods:**

MIR9-3HG, hsa-let-7c-5p, and THBS1 levels were quantified via RT-qPCR in 179 paired HCC tissues and cell lines. Associations between MIR9-3HG expression and clinicopathological features were analyzed using χ^2^ test. The prognostic value was assessed using Kaplan-Meier and Cox regression analyses. The biological functions of MIR9-3HG were evaluated through CCK-8, Transwell assays. The competing endogenous RNA (ceRNA) targeting relationship was validated using dual-luciferase reporter.

**Results:**

MIR9-3HG was significantly upregulated in HCC tissues and cell lines. High MIR9-3HG expression correlated with advanced TNM stage, higher Edmondson-Steiner grade, vascular invasion, and poor overall survival, and was identified as an independent prognostic factor. Hsa-let-7c-5p expression was negatively correlated with MIR9-3HG and THBS1in HCC tissues. In vitro experiments, silencing MIR9-3HG inhibited HCC cell proliferation, migration, and invasion. Luciferase reporter assays demonstrated that MIR9-3HG functioned as a ceRNA to sponge hsa-let-7c-5p. In turn, hsa-let-7c-5p directly targeted and negatively regulated THBS1.

**Conclusion:**

MIR9-3HG promoted HCC aggressiveness by acting as a ceRNA for hsa-let-7c-5p to upregulate THBS1. These findings highlighted its potential as a prognostic biomarker.

## Introduction

Primary liver cancer (PLC) is defined as a malignant tumor originating from hepatocytes or biliary epithelial cells. Globally, its incidence and mortality rank among the most common and lethal malignancies [1–3]. Hepatocellular carcinoma (HCC) represents the predominant subtype of PLC, comprising approximately 80% of all cases. Multiple risk factors contribute to HCC development, including chronic hepatitis of various etiologies and liver cirrhosis, which may eventually progress to liver cancer [4]. HCC is characterized by high malignancy, insidious onset, rapid progression, difficulty in early diagnosis, poor prognosis, and high mortality [5]. Early-stage PLC confined to the liver offers a relatively favorable outcome, with approximately 50% of patients achieving 5‑year survival following curative resection. In contrast, patients with advanced-stage disease who lose the opportunity for surgery have a 5‑year survival rate of less than 20% [6]. Therefore, early detection and screening for liver cancer are of paramount importance.

Various long non-coding RNAs (lncRNAs) and microRNAs (miRNA) have been confirmed to play significant roles in hepatocarcinogenesis and patient prognosis. Several lncRNAs, including BSG-AS1 and HOTTIP, have been validated to be overexpressed in HCC, where their elevated expression is closely linked to poor clinical outcomes [7, 8]. Conversely, lncRNAs such as PWRN1 and HEPFAL were significantly downregulated and correlated with improved prognosis [9, 10]. Aberrant expression of MIR9-3HG has been observed across various cancers, including lung squamous cell carcinoma (LUSC), cervical cancer, head and neck squamous cell carcinoma, and glioma. Functional studies have demonstrated that MIR9-3HG contributed to disease progression through a range of biological processes, including cell proliferation, invasion, apoptosis, epithelial-mesenchymal transition (EMT), DNA methylation, and drug sensitivity [11–14]. For instance, in LUSC, MIR9-3HG promoted tumor progression by sponging miR-138-5p and recruiting TATA-box binding protein 15 (TAF15), thereby regulating both mRNA and protein levels of LIM domain kinase 1 (LIMK1) [15]. Although transcriptomic analyses have suggested an association between MIR9-3HG and immune responses in HCC patients [16], its underlying molecular mechanism in this malignancy remained unexplored.

MiRNAs are endogenous small non‑coding RNAs that typically function by directly degrading mRNA or binding to the 3’UTR of target mRNAs to inhibit translation, ultimately achieving post‑transcriptional regulation of specific protein‑coding genes [17]. Numerous studies have demonstrated that the hsa‑let‑7 family was closely associated with the development of HCC [18–20]. Among them, hsa‑let‑7c‑5p has been extensively reported to act as a tumor suppressor in HCC, inhibiting cancer cell proliferation and migration [21, 22]. Furthermore, hsa‑let‑7c‑5p contributed to sorafenib resistance through modulating cell cycle‑related proteins and related signaling pathway [23, 24]. The diverse biological functions of hsa‑let‑7c‑5p are primarily attributed to its selective regulation of target genes and its dynamic interplay within ceRNA networks in cancer. Additionally, THBS1 has been reported to contribute to HCC progression through various signaling pathways, and has been identified as a key prognostic factor for HCC in Asian populations [25–27].

Although dysregulation of MIR9-3HG has been implicated in multiple cancers, its biological role and clinical significance in HCC remain largely unknown. In this study, we aimed to investigate the expression of MIR9-3HG in HCC tissues and its correlation with clinicopathological characteristics and patient prognosis. Furthermore, we sought to elucidated the molecular mechanism by which the MIR9-3HG/hsa-let-7c-5p/THBS1 axis regulates malignant behaviors of HCC cells.

## Materials and methods

### Clinical data collection

This study enrolled 179 patients with primary HCC who underwent radical resection at Jiaozuo People’s Hospital between September 2018 and January 2021. Paired tumor and adjacent non-tumor tissue samples were obtained from all cases. Specimens were histopathologically confirmed as HCC by expert pathologists. All patients had complete clinical records and had not received any preoperative anti-tumor therapy. Individuals who had undergone surgery within the previous three months or presented with other malignancies were excluded. A five-year postoperative follow-up was conducted, with death or completion of the follow-up period defined as the endpoint events. Preoperative fasting blood samples were collected from all participants to measure serum hepatitis B virus (HBV) and alpha-fetoprotein (AFP) levels. Demographic data (age, gender) and clinical parameters, including liver cirrhosis, tumor size, TNM stage, Edmondson-Steiner (ES) grade, and vascular invasion, were also documented. All experimental procedures strictly adhered to the principles of the Declaration of Helsinki and were approved by the institutional ethics committee. Written informed consent was obtained from each patient and their family members prior to sample collection.

### RT-qPCR

During the experiment, tumor tissues and matched normal liver tissues were transferred to -80 ℃ for long-term storage after quick freezing with liquid nitrogen. Gene expression levels were assessed using RT‑qPCR. Total RNA was extracted with TRIzol Reagent. The qPCR reactions were carried out with Power Track™ SYBR Green Master Mix following the manufacturer’s instructions. Transcript levels of MIR9-3HG and THBS1 were normalized to GAPDH, while U6 served as the endogenous control for hsa-let-7c-5p. These primer sequences were provided in Table 1.

**Table 1:** RT-qPCR primer sequences

Gene	Sequences
LncRNA MIR9-3HG	Forward: 5’-TCTGTTTGGGTCTGAGGTGCATG-3’
	Reverse: 5’-CTATGCAGGGAAAAGGCCCAG-3’
Has-let-7c-5p	Forward: 5’-GCCGAG TGAGGTAGTAGGTTG-3’
	Reverse: 5’-CTGGATACGACGAATTCG-3’
THBS1	Forward: 5’-CACGTGGTGTCTGTGGAAGA-3’
	Reverse: 5’-TAGAGCTGGAGCAGCCTTTG-3’
GAPDH	Forward: 5’-CAGCCTCAAGATCATCAGCA-3
	Reverse: 5’-TGTGGTCATGAGTCCTTCCA-3’
U6	Forward: 5’-CTCGCTTCGGCAGCACA-3’
	Reverse: 5’-AACGCTTCACGAATTTGCGT-3’

### Cell culture and transfection

Human HCC cell lines Huh-7, MHCC97-H, and MHCC97-L were cultured in DMEM. SNU-449 and Li-7 cells were maintained in RPMI 1640 medium. The normal human liver cell line THLE-2 was grown in BEGM supplemented with 5 ng/mL epidermal growth factor (EGF), 70 ng/mL phosphoethanolamine. All medium were supplemented with an additional 10% FBS. All cells were incubated under standard culture conditions. For transfection, cells were seeded into 6-well plates (1 × 10^5^ cells/well) and cultured for 24 h. Transfection mixtures were prepared under sterile conditions. Briefly, Lipofectamine 3000, si-MIR9-3HG, hsa-let-7c-5p mimic, inhibitor and respective negative control (NC) were diluted in Opti-MEM medium separately. The two preparations were combined, gently mixed, and incubated for an additional 20 min at room temperature. Subsequently, the mixtures were added dropwise to the corresponding wells.

### CCK-8 assay

Cell proliferation was assessed using the Cell Counting Kit‑8 (CCK-8) assay. Following trypsinization and counting, cells were plated in 96‑well plates (2 × 10^3^ cells/well), with each well containing 100 µL of culture medium. At designated time points (0, 24, 48, 72, and 96 h), the medium was aspirated and replaced with same volume of CCK8 working solution. Absorbance (OD_450_) was measured using a microplate reader after these plates were incubated in the dark for 1–2 h.

### Transwell assays

Cell migratory and invasive capacities were assessed using Transwell assays. For invasion assays, inserts pre‑coated with mrigel were employed. Cells were trypsinized and resuspended in serum-free medium. 250 µL of the cell suspension, containing approximately 3 × 10^5^ cells, was seeded into the upper chamber. Subsequently, the lower chamber was filled with 700 µL of medium containing 10% FBS, followed by incubation of the cells under standard culture conditions for 24 h. Following incubation, cells that had migrated or invaded were fixed by adding 700 µL of 4% paraformaldehyde, stained with 700 µL of 0.2% crystal violet. Stained cells were imaged and counted in five random fields per well under a microscope.

### Dual-luciferase reporter assay

To construct the ceRNA network, potential lncRNA-miRNA interactions were first predicted using the starBase database. Subsequently, miRNA-mRNA interactions were predicted using bioinformatics databases: TargetScan, miRDB, StarBase, and miRWalk. The overlapping mRNA targets were visualized using Venny 2.1.0. Based on these predictions, wild‑type (WT) and mutant (MUT) dual‑luciferase reporter vector of MIR9-3HG and THBS1 containing the predicted binding sites were constructed. For the reporter assay, cells were co‑transfected with either WT or MUT reporter plasmids and hsa‑let‑7c‑5p mimic or inhibitor. After 48 h, luciferase activity was measured.

### Statistical analysis

Group comparisons were using Student’s t‑test, one-way ANOVA, two-way ANOVA, or χ^2^ test, as appropriate, with SPSS 23.0 and GraphPad Prism 9.0. For the lncRNA MIR9-3HG analyzed herein, its measured values were dichotomized using the median as the threshold. Cases at or above the median formed the high-level group, whereas those below the median constituted the low-level group. Applying the median as the dividing criterion guaranteed equal numbers across the two groups, kept the grouping procedure objective, and required no assumption of any parametric distribution. Overall survival was estimated using the Kaplan-Meier (K-M) method, and independent prognostic factors were determined using multivariate Cox regression. Pearson’s correlation analysis was performed to evaluate the correlations among MIR9‑3HG, hsa‑let‑7c‑5p, and THBS1 expression. *p* < 0.05 was defined as statistically significant.

## Results

### Differences in MIR9-3HG expression between tumor and adjacent tissues and its impact on patient survival

**Fig. 1:**
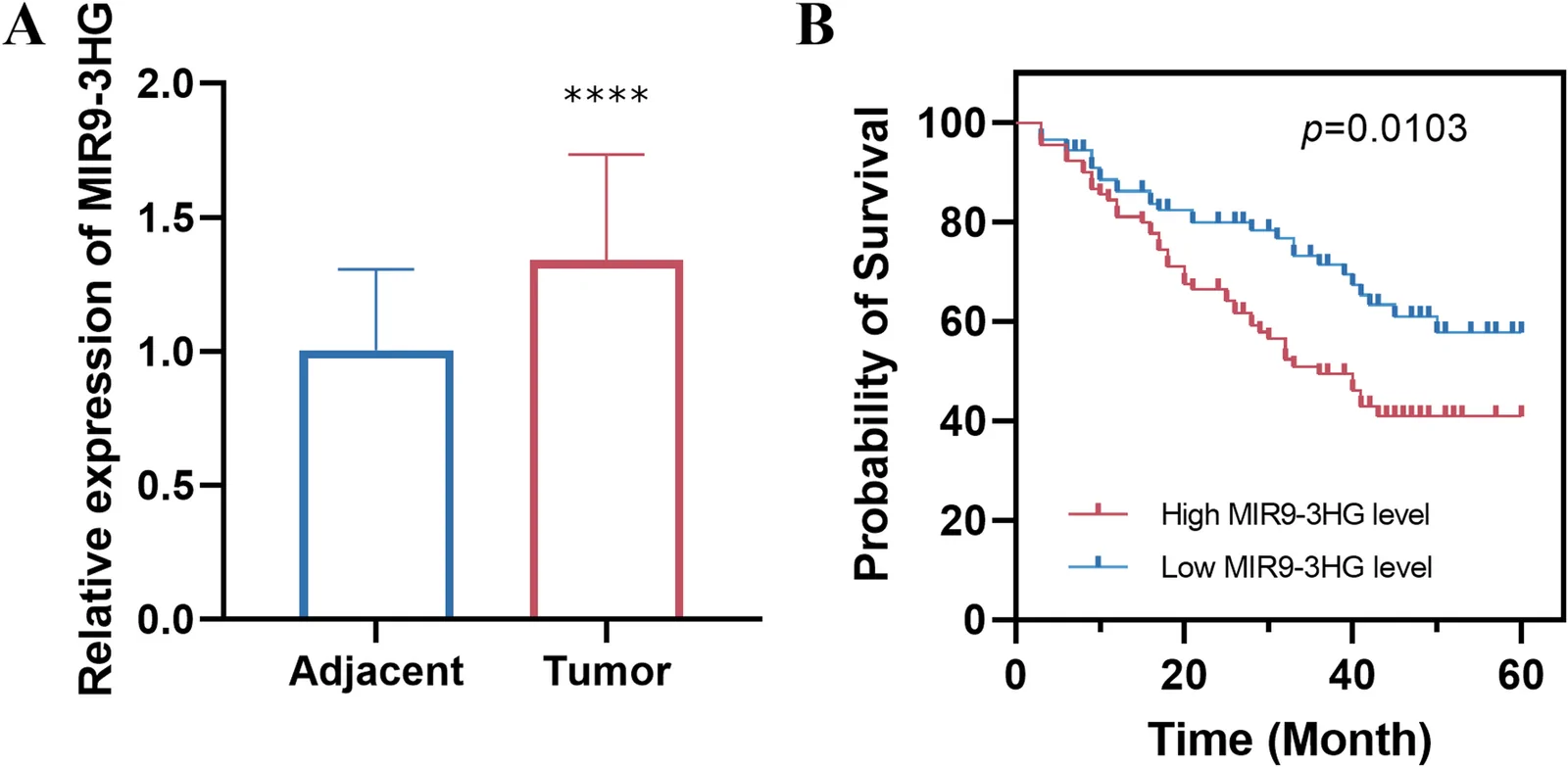
MIR9-3HG expression was upregulated and correlated with poor prognosis in HCC. **A** MIR9-3HG levels in HCC specimens and matched adjacent non‑tumor tissues. **B** K-M survival curves for overall survival of 179 HCC patients stratified by MIR9-3HG expression (high vs. low, based on the median expression). **** means *p* < 0.0001

The relative expression level of MIR9-3HG was markedly elevated in HCC tissues than in paired adjacent non-tumor tissues (*p* < 0.0001, Fig. 1A). To assess the prognostic value of MIR9-3HG, K-M survival curves were generated which revealed that overall survival was significantly poorer in patients with high MIR9-3HG expression than in those with low expression (*p* = 0.0103, Fig. 1B). These findings indicated that high MIR9-3HG levels might be associated with worse clinical outcomes in HCC patients.

### MIR9-3HG expression correlated with HCC clinicopathological characteristics

Using the median expression level of MIR9-3HG as the cutoff, 179 HCC patients were divided into high (*n* = 90) and low (*n* = 89) expression groups, and the correlation between MIR9-3HG expression and various clinicopathological characteristics was evaluated (Table 2). Elevated MIR9-3HG expression was significantly correlated with aggressive tumor features. Specifically, the high-expression group exhibited a greater proportion of patients with advanced TNM stage (III-IV: 35.56% vs. 20.22%, *p* = 0.022), higher ES grade (III-IV: 31.11% vs. 15.73%, *p* = 0.015), and the presence of vascular invasion (15.56% vs. 5.62%, *p* = 0.031). In contrast, MIR9-3HG levels had no correlation with other clinicopathological Characteristics (all *p* > 0.05).

**Table 2:** Association of MIR9-3HG expression levels with clinical characteristics in HCC patients

Characteristics	Total (*n* = 179)	MIR9-3HG expression levels		*p*
		High (*n* = 90)	Low (*n* = 89)	
Age (years)				
< 55	81	40 (44.44)	41 (46.07)	0.827
≥ 55	98	50 (55.56)	48 (53.93)	
Gender				
Male	118	61 (67.78)	57 (64.04)	0.598
Female	61	29 (32.22)	32 (35.96)	
HBV infection				
No	56	26 (28.89)	30 (33.71)	0.487
Yes	123	64 (71.11)	59 (66.29)	
AFP (ng/ml)				
< 400	110	53 (58.89)	57 (64.04)	0.479
≥ 400	69	37 (41.11)	32 (35.96)	
Liver cirrhosis				
Without	66	31 (34.44)	35 (39.33)	0.499
With	113	59 (65.56)	54 (60.67)	
Tumor size (cm)				
< 5	84	39 (43.33)	45 (50.56)	0.333
≥ 5	95	51 (56.67)	44 (49.44)	
TNM stage				
I-II	129	58 (64.44)	71 (79.78)	**0.022**
III-Ⅳ	50	32 (35.56)	18 (20.22)	
ES grade				
I-II	137	62 (68.89)	75 (84.27)	**0.015**
III-Ⅳ	42	28 (31.11)	14 (15.73)	
Tumor number				
Single	120	58 (64.44)	62 (69.66)	0.458
Multiple	59	32 (35.56)	27 (30.34)	
Vascular invasion				
No	160	76 (84.44)	84 (94.38)	**0.031**
Yes	19	14 (15.56)	5 (5.62)	

*HCC* Hepatocellular Carcinoma, *HBV* Hepatitis B Virus, *AFP* Alpha-fetoprotein, *TNM stage* Tumor-Node-Metastasis stage system, *ES grade* Edmondson-Steiner grade

Data presented in bold font correspond to *P* < 0.05, which indicates statistically significant differences among groups

### Multivariate Cox regression analysis of prognostic factors for HCC overall survival

To identify the prognostic factors for overall survival in HCC patients, a multivariate cox proportional hazards regression analysis was conducted for the clinicopathological indicators and MIR9-3HG expression levels of 179 enrolled HCC patients (Table 3). In the survival analysis, a Cox proportional hazards regression model was employed. All baseline variables presented in Table 2 were simultaneously forced into a multivariable model via the Enter method to fully appraise their independent prognostic values. The resultant HR and associated 95% CI were reported for every variable. The results revealed that TNM stage (HR = 1.811, 95% CI: 1.074–3.054, *p* = 0.026), ES grade (HR = 1.813, 95% CI: 1.058–3.105, *p* = 0.030), and vascular invasion (HR = 1.945, 95% CI: 1.030–3.674, *p* = 0.040) were independently associated with poor overall survival. Notably, after adjusting for potential confounders, high MIR9-3HG expression remained a significant predictor of reduced survival (HR = 2.027, 95% CI: 1.208–3.401, *p* = 0.007). In contrast, other factors (HBV infection, tumor size, tumor number, etc.) did not show independent prognostic value in the multivariable model (all *p* > 0.05).

**Table 3:** Multivariable Cox analysis of factors associated with overall survival in HCC patients

Factors	Multivariate analysis		
	*p*	HR	95%CI
Age	0.408	1.233	0.751–2.024
Gender	0.590	1.158	0.680–1.972
HBV infection	0.210	1.391	0.830–2.331
AFP	0.210	1.394	0.829–2.344
Liver cirrhosis	0.239	1.328	0.828–2.132
Tumor size	0.077	1.574	0.952–2.604
TNM stage	**0.026**	1.811	1.074–3.054
Edmondson-Steiner grade	**0.030**	1.813	1.058–3.105
Tumor number	0.104	1.492	0.921–2.419
Vascular invasion	**0.040**	1.945	1.030–3.674
LncRNA MIR9-3HG	**0.007**	2.027	1.208–3.401

*HCC* Hepatocellular Carcinoma, *HR* Hazard ratio, *CI* Confidence interval, *HBV* Hepatitis B Virus, *AFP* Alpha-fetoprotein, *TNM stage* Tumor-Node-Metastasis stage system, *ES grade* Edmondson-Steiner grade

Data presented in bold font correspond to *P* < 0.05, which indicates statistically significant differences among groups

### Expression of MIR9-3HG in HCC cell lines and its effects on malignant phenotypes

**Fig. 2:**
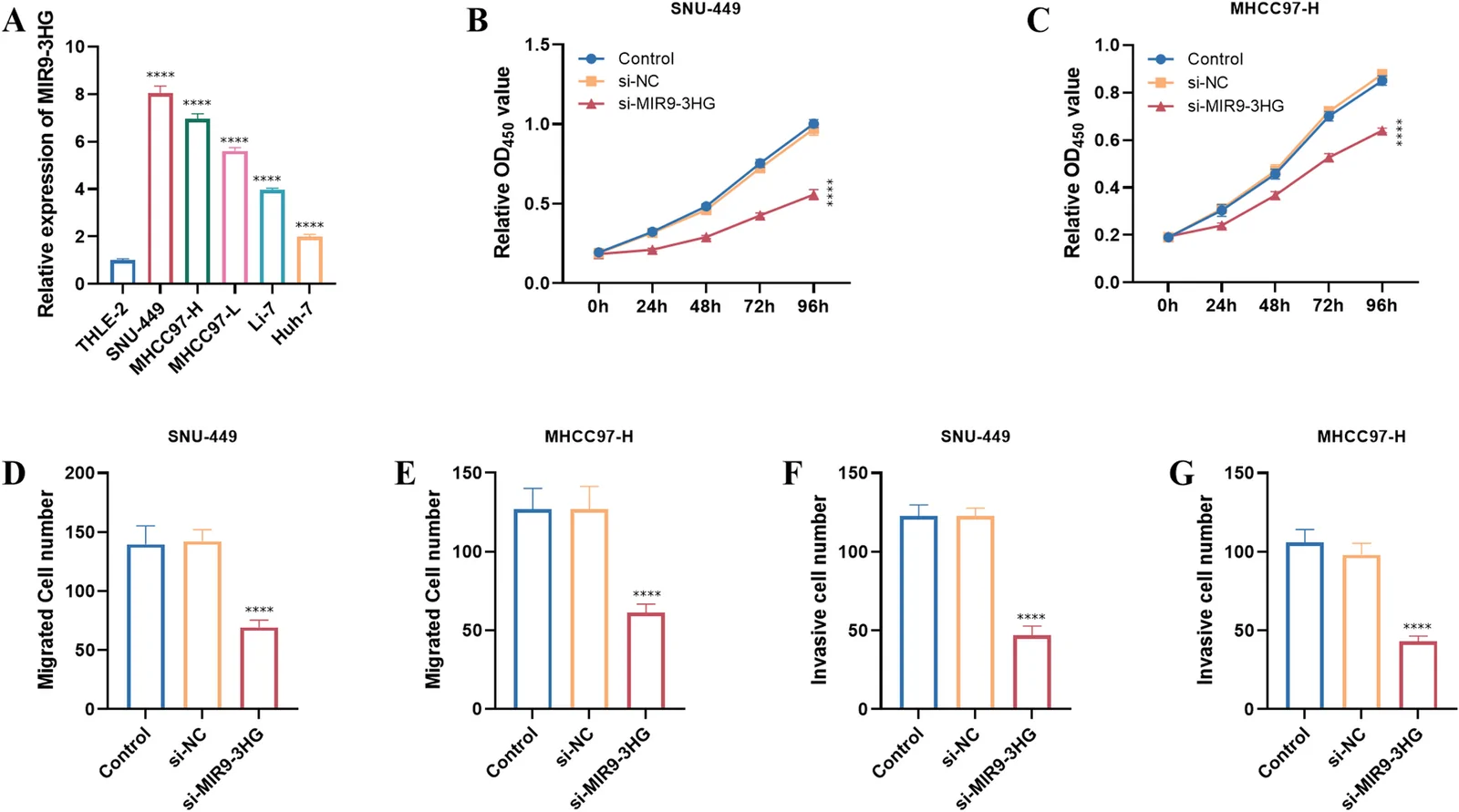
MIR9-3HG knockdown inhibited proliferation, migration, and invasion of HCC cells in vitro. **A** MIR9-3HG expression in HCC cell lines and the normal hepatic cell line THLE-2. **B**,** C** CCK-8 assays showing cell proliferation was significantly suppressed in MIR9-3HG knockdown group in SNU‑449 (**B**) and MHCC97‑H (**C**) cells. **D**,** E** Migration assays demonstrating a marked reduction in migrated cell numbers following MIR9-3HG knockdown in SNU‑449 (**D**) and MHCC97‑H (**E**) cells. **F**,** G** Invasion assays revealing a significant decrease in invasive cell numbers in MIR9-3HG knockdown in SNU‑449 (**F**) and MHCC97‑H (**G**) cells. **** means *p* < 0.0001. The quantitative data were derived from microscopic images with a scale bar (100 μm); only the bar graph is presented in this figure. All the cell function experiments were independently replicated at least three times, and in each independent replication, three technical replicates were set up

To further investigate the biological functions of MIR9-3HG, we examined the relative expression of MIR9-3HG in normal hepatocyte cell line THLE-2 and multiple HCC cell lines (SNU-449, MHCC97-H, MHCC97-L, Li-7, Huh-7). Compared with THLE-2 cells, MIR9-3HG was noticeably upregulated in all tested HCC cell lines (*p* < 0.0001, Fig. 2A). To further explore its biological role, we selected SNU-449 and MHCC97-H cell lines, both of which exhibited high MIR9-3HG expression. Subsequently, MIR9-3HG expression was knocked down using small interfering RNA (siRNA). CCK-8 assays showed that, compared with the si-NC groups, knockdown of MIR9-3HG significantly suppressed the proliferation rate of SNU-449 (Fig. 2B) and MHCC97-H (Fig. 2C) cells within 24 to 96 h (*p* < 0.0001). These results indicated that inhibition of MIR9-3HG expression can suppress the in vitro proliferation of HCC cells. Migration assays were then performed. In both SNU-449 (Fig. 2D) and MHCC97-H (Fig. 2E) cells, the number of migrated cells in the si-MIR9-3HG group was markedly lower than that in the control groups (*p* < 0.0001). Meanwhile, Invasion assays revealed that silencing MIR9-3HG produced a similar trend to that observed in migration assays. In both SNU-449 (Fig. 2F) and MHCC97-H (Fig. 2G) cells, the number of invasive cells in the si-MIR9-3HG group was significantly decreased compared with the control groups (*p* < 0.0001). These demonstrated that silencing MIR9-3HG expression can inhibit the in vitro migration and invasion ability of HCC cells. These findings indicated that MIR9-3HG might act as an oncogene in HCC by promoting proliferation, migration, and invasion.

### MIR9-3HG targeted and regulated hsa-let-7c-5p

**Fig. 3:**
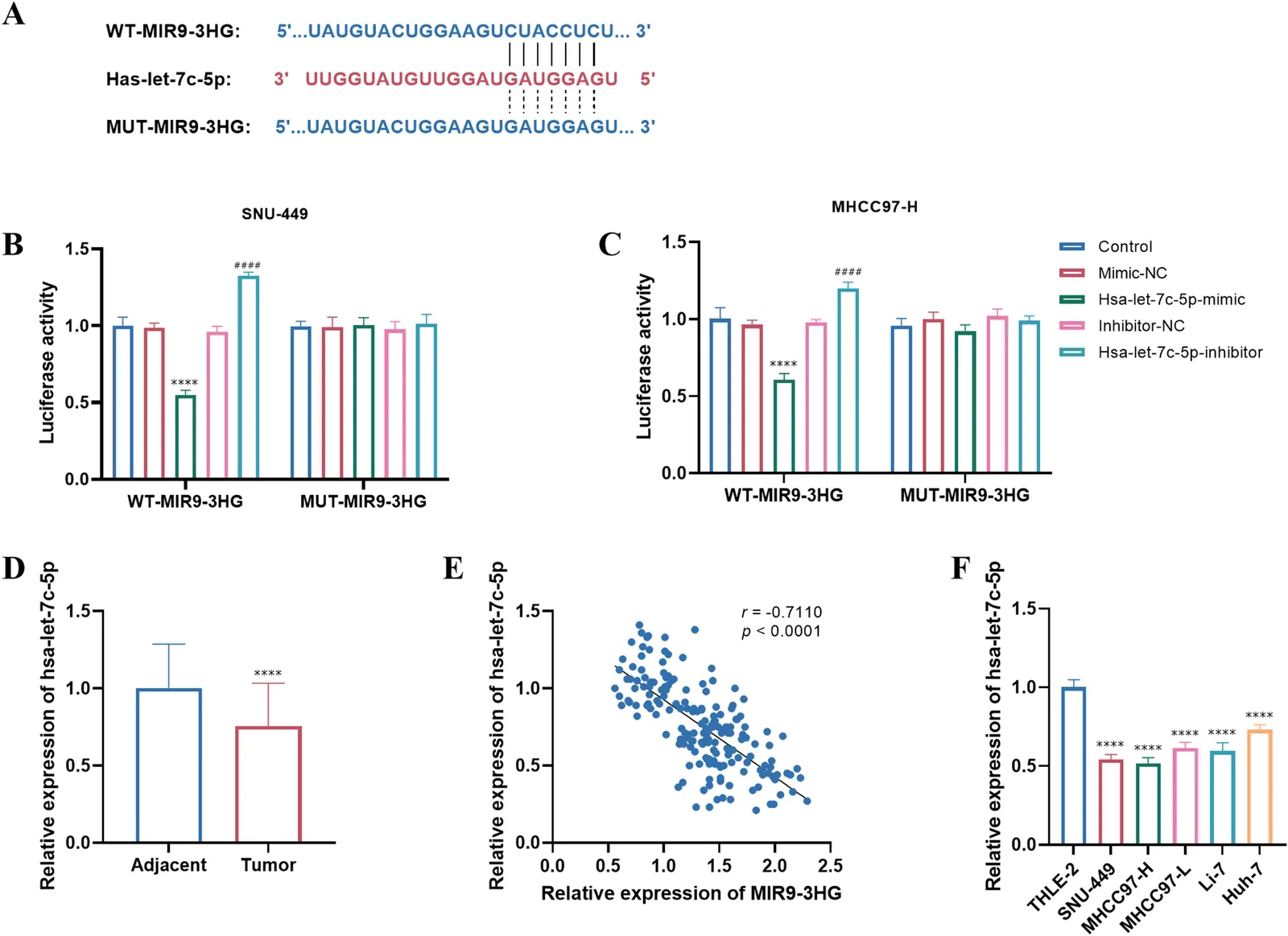
MIR9-3HG functioned as a molecular sponge for hsa-let-7c-5p. **A** Putative binding sites between MIR9-3HG and hsa-let-7c-5p. **B**,** C** Luciferase activity assays in SNU-449 (**B**) and MHCC97-H (**C**) cells following co‑transfection with wild‑type (WT) or mutant (MUT) MIR9-3HG reporter plasmids together with hsa-let-7c-5p mimic or inhibitor, **** means hsa-let-7c-5p mimic vs. mimic-NC *p* < 0.0001, #### means hsa-let-7c-5p-inhibitor vs. inhibitor-*NC p* < 0.0001. **D** hsa-let-7c-5p expression levels in HCC specimens and paired adjacent non‑tumor tissues, **** means *p* < 0.0001 **E** Correlation between MIR9-3HG and hsa-let-7c-5p expression in HCC tissues. **F** hsa-let-7c-5p expression in HCC cell lines and the normal hepatic cell line THLE-2, **** means *p* < 0.0001. All the cell function experiments were independently replicated at least three times, and in each independent replication, three technical replicates were set up

The starBase database predicted potential complementary binding sites between lncRNA MIR9-3HG and hsa-let-7c-5p (Fig. 3A). Dual-luciferase reporter assays revealed that, in SNU-449 (Fig. 3B) and MHCC97-H (Fig. 3C) cells, co-transfection with hsa-let-7c-5p mimic significantly reduced the luciferase activity of the WT-MIR9-3HG vector (*p* < 0.0001) compared with the control group, while no obvious change was observed in the MUT-MIR9-3HG vector. These results confirmed that MIR9-3HG might interact with hsa-let-7c-5p via the targeted binding sites. We further detected hsa-let-7c-5p levels in HCC tissues and found that its levels were markedly lower in tumor tissues than in paired adjacent non-tumor tissues (*p* < 0.0001, Fig. 3D). And a significant inverse correlation was observed between MIR9-3HG and hsa-let-7c-5p expression in HCC specimens (*r*=-0.7110, *p* < 0.0001, Fig. 3E). In addition, hsa-let-7c-5p expression was decreased in various HCC lines compared to normal hepatocyte cell line THLE-2 (Fig. 3F). These results suggested that MIR9-3HG had a negative targeting relationship with hsa-let-7c-5p.

### MIR9-3HG facilitated HCC malignant phenotypes by sponging hsa-let-7c-5p

**Fig. 4:**
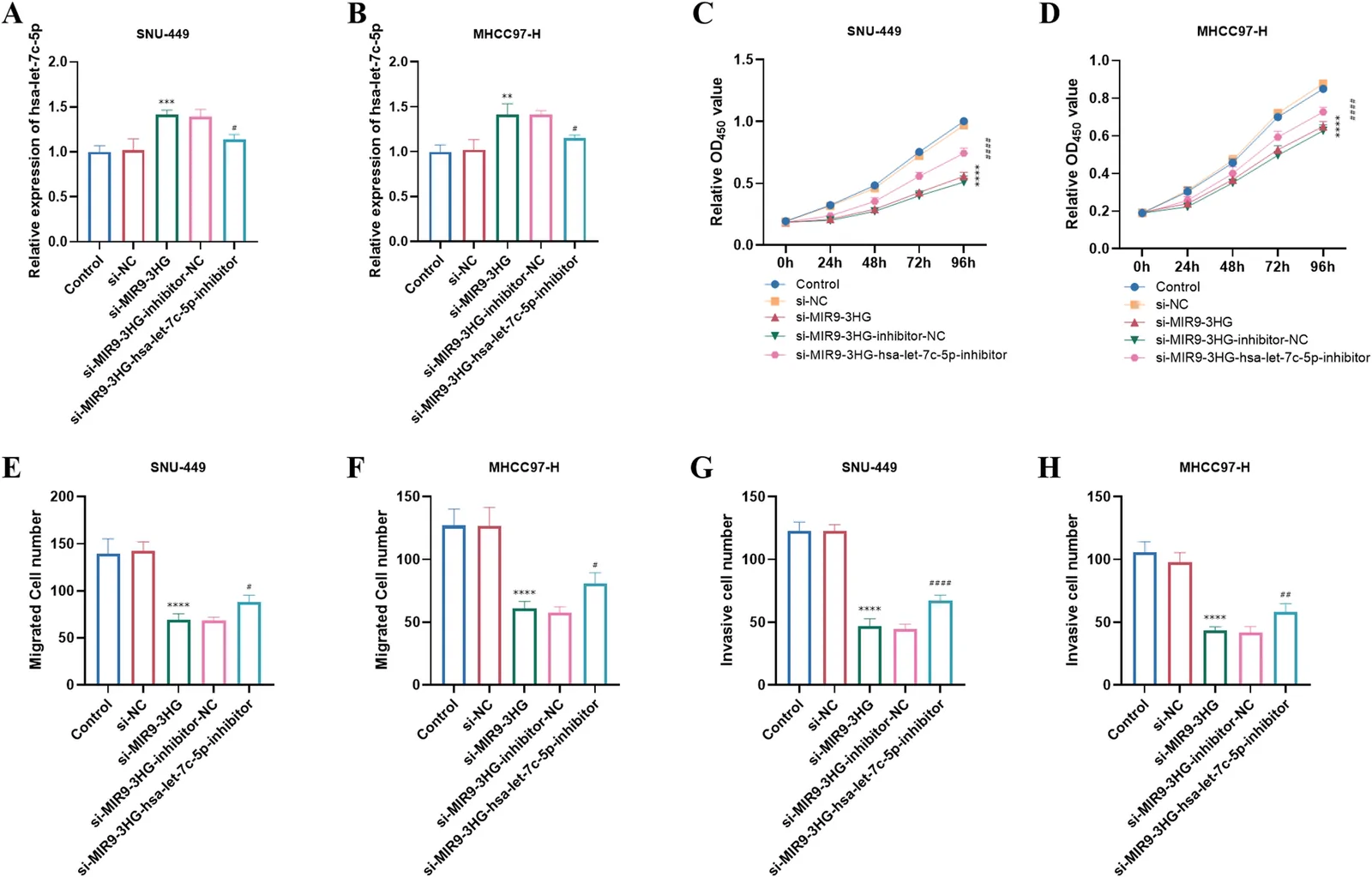
MIR9-3HG regulated cell proliferation, migration and invasion by sponging hsa-let-7c-5p in vitro. **A**,** B** hsa-let-7c-5p expression was significantly increased upon MIR9-3HG knockdown in SNU-449 (**A**) and MHCC97-H (**B**) cells, and this effect was partially abrogated by co‑transfection with hsa-let-7c-5p inhibitor. **C**,** D** CCK-8 assays showing the suppression of cell proliferation induced by MIR9-3HG knockdown in SNU-449 (**C**) and MHCC97-H (**D**) cells was partially rescued by hsa-let-7c-5p inhibition. **E**,** F** Migration assays demonstrating the reduction in migrated cell numbers following MIR9-3HG knockdown in SNU-449 (**E**) and MHCC97-H (**F**) cells was partially reversed upon co‑transfection with hsa-let-7c-5p inhibitor. **G**,** H** Invasion assays revealing the decreased invasive cell numbers resulting from MIR9-3HG depletion in SNU-449 (**G**) and MHCC97-H (**H**) cells was partially restored by hsa-let-7c-5p inhibitor co‑transfection. * Means si-MIR9-3HG vs. si-NC, # means si-MIR9-3HG-hsa-let-7c-5p inhibitor vs. si-MIR9-3HG-inhibitor-NC, **** or #### means *p* < 0.0001, ***means *p* < 0.001, ** or ## means *p* < 0.01, # means *p* < 0.05. The quantitative data were derived from microscopic images with a scale bar (100 μm); only the bar graph is presented in this figure. All the cell function experiments were independently replicated at least three times, and in each independent replication, three technical replicates were set up

To determine whether MIR9-3HG exerted its oncogenic role by negatively regulating hsa-let-7c-5p, rescue experiments were performed in SNU-449 and MHCC97-H cells via transfection with hsa-let-7c-5p inhibitor. Knockdown of MIR9-3HG led to a marked increase in hsa-let-7c-5p levels, an effect that was partially reversed upon introduction of the hsa-let-7c-5p inhibitor (Fig. 4A-B). Functional assays revealed that the suppression of HCC cell proliferation, migration, and invasion by MIR9-3HG silencing was largely reversed following hsa-let-7c-5p inhibitor transfection (Fig. 4C-H). Collectively, these findings suggested that MIR9-3HG may promote malignant behaviors in HCC cells by sponging hsa-let-7c-5p, thereby relieving its repressive impact on downstream target genes.

### MIR9-3HG regulated THBS1 expression by sponging hsa-let-7c-5p

**Fig. 5:**
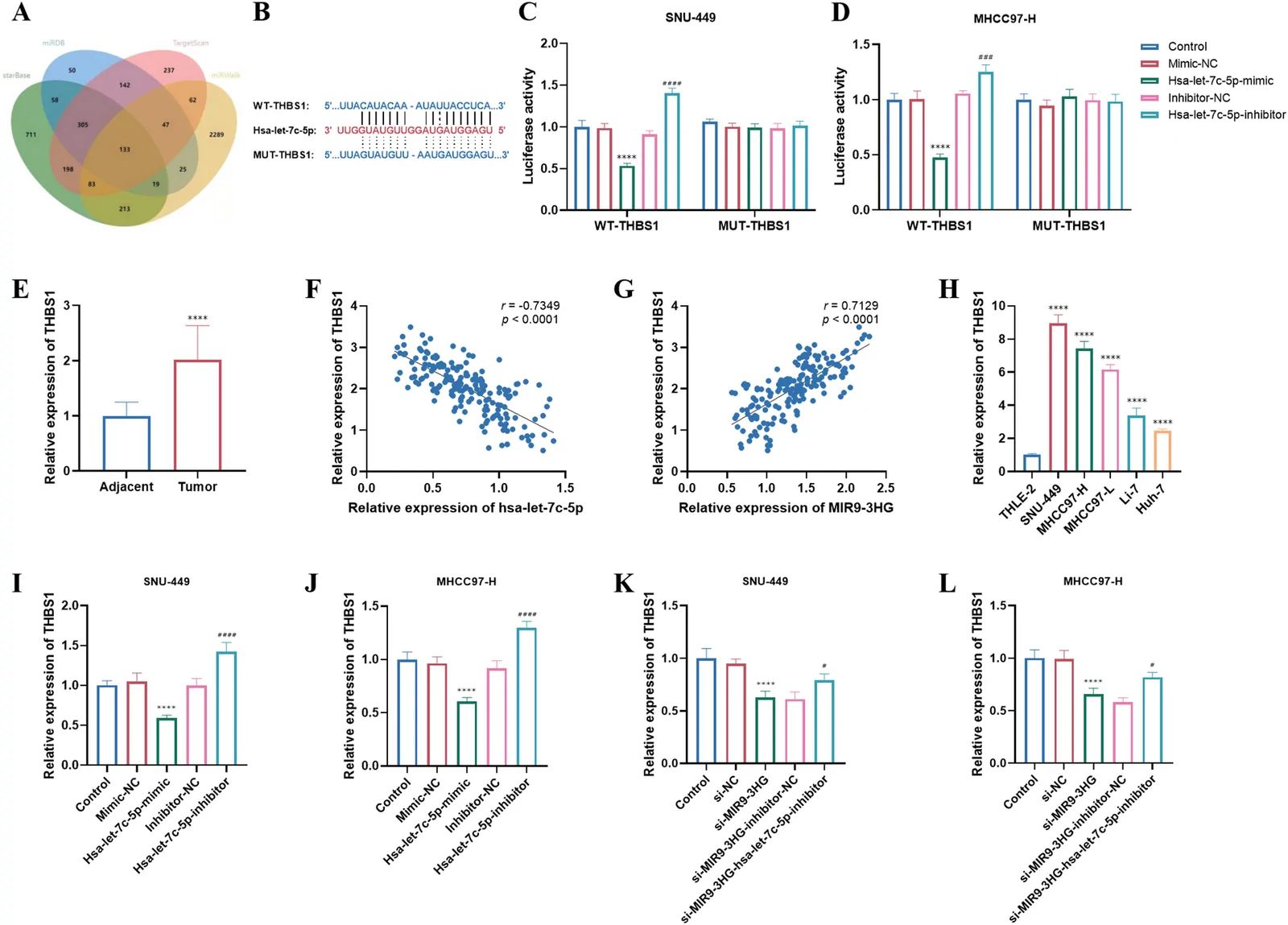
MIR9-3HG upregulated THBS1 by sponging hsa-let-7c-5p. **A** Venny diagram showed the number of target genes with predicted binding sites with hsa-let-7c-5p. **B** Putative binding sites between hsa-let-7c-5p and THBS1. **C**,** D** Luciferase activity assays in SNU-449 (**C**) and MHCC97-H (**D**) cells following co‑transfection with wild‑type (WT) or mutant (MUT) THBS1 reporter plasmids together with hsa-let-7c-5p mimic or inhibitor, **** means hsa-let-7c-5p mimic vs. mimic-NC *p* < 0.0001, #### means hsa-let-7c-5p-inhibitor vs. inhibitor-NC *p* < 0.0001, ### means *p* < 0.001. **E** THBS1 expression levels in HCC specimens and paired adjacent non‑tumor tissues, **** means *p* < 0.0001. **F** Correlation between hsa-let-7c-5p and THBS1 expression in HCC tissues. **G** Correlation between MIR9-3HGand THBS1 expression in HCC tissues. **H** THBS1 expression in HCC cell lines and the normal hepatic cell line THLE-2, **** means *p* < 0.0001. **I**,** J** Overexpression of hsa-let-7c-5p markedly downregulated THBS1, while inhibition of hsa-let-7c-5p upregulated it SNU-449 (**I**) and MHCC97-H (**J**)cells respectively, **** means hsa-let-7c-5p mimic vs. mimic-NC *p* < 0.0001, #### means hsa-let-7c-5p-inhibitor vs. inhibitor-NC *p* < 0.0001. **K**,** L** MIR9-3HG knockdown significantly reduced THBS1 expression, which was partially restored by co‑transfection with hsa-let-7c-5p inhibitor in SNU-449 (**K**) and MHCC97-H (**L**) cells, **** means si-MIR9-3HG vs. si-NC *p* < 0.0001, # means si-MIR9-3HG-let-7c-5p inhibitor vs. si-MIR9-3HG-inhibitor-NC *p* < 0.05. All the cell function experiments were independently replicated at least three times, and in each independent replication, three technical replicates were set up

To identify the potential downstream targets of hsa-let-7c-5p, four bioinformatics databases (TargetScan, miRDB, starBase, and miRWalk) were used. Based on prediction scores ranked from highest to lowest, a Venn diagram analysis identified THBS1 as a candidate target across multiple databases, placing it among the top ten of 133 overlapping targets (Fig. 5A), suggesting it may be a reliable downstream target of it. Sequence alignment in starBase database further confirmed specific complementary binding sites between them (Fig. 5B). To validate direct interaction, dual luciferase reporter vectors containing wild-type-THBS1 and mutant-THBS1 target sites were generated. In SNU-449 and MHCC97-H cells, overexpression of hsa-let-7c-5p (hsa-let-7c-5p-mimic) led to a marked decrease in luciferase activity from the WT-THBS1 vector (*p* < 0.0001), while no obvious inhibitory effect was observed on the MUT-THBS1 vector. Conversely, inhibition of hsa-let-7c-5p markedly enhanced luciferase activity of the WT-THBS1 vector (*p* < 0.001), confirming their targeted binding relationship (Fig. 5C-D).

Expression analysis showed that THBS1 was upregulated in HCC tissues (*p* < 0.0001, Fig. 5E). Correlation analysis revealed that THBS1 expression was negatively correlated with hsa-let-7c-5p levels (*r* = -0.7349, *p* < 0.0001, Fig. 5F), while positively correlated with MIR9-3HG (*r* = 0.7129, *p* < 0.0001, Fig. 5G). At the cell line level, THBS1 levels were markedly higher in HCC cell lines (Fig. 5H). To clarify the regulatory relationship among MIR9-3HG, hsa-let-7c-5p, and THBS1, we performed functional experiments. Overexpression of hsa-let-7c-5p significantly downregulated THBS1 expression (*p* < 0.0001), whereas inhibition of hsa-let-7c-5p led to a marked upregulation (*p* < 0.0001, Figs. 5I-J). Furthermore, knockdown of MIR9-3HG reduced THBS1 expression, an effect that was partially reversed upon co-transfection with hsa-let-7c-5p inhibitor (*p* < 0.0001, Figs. 5K-L). Taken together, these findings indicated that MIR9-3HG might modulate THBS1 expression by competitively binding to hsa-let-7c-5p, which abrogated the suppressive effect on this target.

## Discussion

To date, investigation into the MIR9-3HG within HCC remains at an early stage. A systematic literature search has retrieved only a handful of directly or indirectly relevant reports. One such study, a bioinformatics analysis published in 2020, identified MIR9-3HG as an immune-related lncRNA through integrated computational approaches. That work reported upregulation of this lncRNA in LIHC tissues and stratified LIHC patients into three groups based on immune-related risk profiles. Notably, LIHC patients harboring a more abundant immune cell infiltrate experienced better outcomes [16]. However, this investigation was purely computational, lacking any functional validation via in vivo or in vitro experiments, and did not explore the downstream mechanisms underlying MIR9-3HG action. Overall, existing evidence suffers from multiple deficiencies, the specific ceRNA network downstream of MIR9-3HG remains unverified experimentally; the key target genes and signaling pathways modulated by this lncRNA have not been identified; and the relationship between MIR9-3HG expression and clinicopathological features has not been systematically assessed. Accordingly, a comprehensive dissection of the functional mechanisms of MIR9-3HG in HCC, along with elucidation of its precise role within the ceRNA network, represents a pressing scientific challenge that awaits resolution.

This study reports several new findings regarding MIR9-3HG in HCC. First, we demonstrate for the first time paired HCC tissues that MIR9-3HG is markedly upregulated. High expression of this lncRNA independently correlates with advanced tumor stage, higher Edmonson-Steiner grade, vascular invasion, and shortened overall survival, thus systematically extending and validating prior clinical evidence. While MIR9-3HG has been shown to drive LUSC tumorigenesis by sponging miR-138-5p to elevate LIMK1 [15], we now provide evidence that it also acts as an oncogene in HCC. More importantly, we uncover a previously unknown mechanism. MIR9-3HG promotes HCC cell proliferation, migration, and invasion through competitive binding of hsa-let-7c-5p, which in turn relieves THBS1 from repression. Relative to current clinical practice using lenvatinib combined with immunotherapy and locoregional therapy a strategy with promising real-world efficacy [28] yet still limited by high-grade adverse events like thrombotic thrombocytopenic purpura our discovery supports a precision medicine approach targeting a specific molecule, potentially complementing combination regimens. From a methodological perspective, while we also combine computational biology with experimental validation similar to network pharmacology and molecular docking studies [29, 30], those studies primarily explore upstream active components from drugs or herbal compounds. In contrast, we start from an endogenous lncRNA and comprehensively delineate a 3′UTR signaling axis governed by ceRNA regulation. This work addresses the knowledge gap concerning MIR9-3HG’s role in the HCC ceRNA network and furnishes multi-faceted evidence for its candidacy as an early diagnostic marker and therapeutic target.

Early diagnosis of HCC remains a global challenge. AFP is currently the most widely used clinical biomarker for HCC detection; however, its sensitivity and specificity are suboptimal. A considerable proportion of HCC patients present with normal AFP levels. Moreover, AFP levels can be influenced by hepatic inflammation, necrosis, and regeneration, leading to elevated AFP in some individuals without malignant transformation. Therefore, identifying novel biomarkers for prognostic assessment of HCC, elucidating their underlying molecular mechanisms, and discovering potential therapeutic targets represent pressing priorities [31]. Therefore, identifying novel biomarkers for early diagnosis and prognostic assessment of HCC, elucidating their underlying molecular mechanisms, and discovering potential therapeutic targets represent pressing priorities. Although previous studies have established associations between MIR9-3HG and various malignancies, its precise role in tumorigenesis and disease progression remained poorly understood, with many aspects yet to be elucidated. In particular, the relationship between MIR9-3HG and HCC has rarely been investigated. In this study, analysis of paired tissue samples revealed that MIR9-3HG levels were markedly elevated in HCC tissues. Notably, higher MIR9-3HG levels were correlated with poorer overall survival in HCC patients and acted as an independent prognostic factor. Its expression levels were also associated with tumor stage, ES grade, and vascular invasion. Collectively, these findings suggested that MIR9-3HG held promise as a potential biomarker for early diagnosis and prognosis prediction in HCC, warranting further clinical validation.

Furthermore, our experimental results demonstrated that MIR9-3HG levels were markedly upregulated in HCC cell lines. Functional cellular assays revealed that knockdown of MIR9-3HG substantially suppressed the proliferation, migration, and invasion of HCC cells. Collectively, these findings suggested that MIR9-3HG played a contributory role in hepatocarcinogenesis and tumor progression. This observation was consistent with previously reported oncogenic roles of MIR9‑3HG in LUSC and cervical cancer [11, 15].

The ceRNA mechanism, by which lncRNAs sponge miRNAs to regulate target gene expression, played a pivotal role in HCC [32]. Bioinformatics analysis predicted potential complementary binding sites between MIR9-3HG and members of the hsa-let-7 family, with multiple databases specifically identifying hsa-let-7c-5p as a candidate interacting miRNA. Single-cell sequencing data revealed that hsa-let-7c-5p levels were markedly reduced in HCC tissues compared to non-cancerous tissues, and its expression was linked to patient prognosis [33]. Previous studies have demonstrated that its overexpression attenuated the proliferative, migratory, and invasive capabilities of HCC cells [21, 34]. In the present research, dual-luciferase reporter assays confirmed that MIR9-3HG may function as a molecular sponge for hsa-let-7c-5p. A negative correlation between their expression levels was observed in HCC tissues, and knockdown of MIR9-3HG substantially upregulated its expression, further validating their targeted interaction. Our findings demonstrated that let-7c-5p expression was downregulated in both HCC tissues and cell lines. Furthermore, rescue experiments confirmed that its inhibition partially reversed the suppressive effects on HCC cell proliferation, migration, and invasion induced by MIR9-3HG knockdown. These results were consistent with previous reports establishing hsa-let-7c-5p as a tumor suppressor in various malignancies [35].

THBS1 expression in malignant cells is regulated by both oncogenes and tumor suppressors, and plays a critical role in tumor progression and metastasis. Additionally, THBS1 restricts antitumor immunity through CD47‑dependent modulation of innate and adaptive immune cells [36]. Emerging evidence has linked THBS1 expression to survival outcomes in HCC patients [37]. In the present study, we focused on the regulatory relationship between hsa‑let‑7c‑5p and THBS1 in HCC. Using bioinformatic prediction (TargetScan, miRDB, StarBase, and miRWalk) combined with dual‑luciferase reporter assay, we validated a direct targeting interaction between hsa‑let‑7c‑5p and the 3′UTR of THBS1. To our knowledge, this is the first report of a direct hsa‑let‑7c‑5p/THBS1 axis in HCC. Correlation analysis unveiled a negative association between their expression in HCC tissues. Notably, THBS1 levels were markedly elevated in HCC specimens and exhibited a strong positive correlation with MIR9‑3HG expression. Functional assays demonstrated that inhibition of hsa‑let‑7c‑5p upregulated THBS1 expression, whereas overexpression of hsa‑let‑7c‑5p or knockdown of MIR9‑3HG substantially reduced THBS1. Taken together, these findings suggested that MIR9‑3HG may upregulate THBS1 by competitively sponging hsa‑let‑7c‑5p, thereby promoting proliferation and migration of HCC cells and contributing to tumor progression. This proposed mechanism offered new insights into the molecular basis of HCC development.

The present study has several limitations that should be acknowledged. First, our findings were primarily based on in vitro experiments demonstrating that MIR9-3HG functioned as a ceRNA by sponging hsa-let-7c-5p, thereby upregulating THBS1 levels. Future investigations incorporating in vivo nude mouse models are warranted to validate this regulatory mechanism. Additionally, larger cohort studies encompassing diverse ethnic and geographical populations will be necessary to firmly establish the role of the MIR9-3HG/hsa-let-7c-5p/THBS1 axis in HCC progression. Second, the upstream regulators and downstream signaling pathways involved in this axis remain to be fully elucidated.

In conclusion, we identified that MIR9-3HG was markedly upregulated in HCC tissues and its higher expression was associated with poor clinical prognosis in HCC patients based on our cohort analysis. In vitro functional assays demonstrated that MIR9-3HG promoted proliferation, migration, and invasion of HCC cells, while its silencing attenuated these malignant phenotypes. Mechanistically, we found that MIR9-3HG acted as a ceRNA by sponging hsa-let-7c-5p, leading to subsequent upregulation of THBS1. These findings suggest a potential pro-tumorigenic role of the MIR9-3HG/hsa-let-7c-5p/THBS1 axis in HCC cell models. However, it is important to note that in vitro results may not fully recapitulate the complex tumor microenvironment in vivo, and the direct extrapolation of these findings to clinical prognosis or diagnostic application requires further validation using independent large-scale clinical cohorts, functional animal models, and rigorous assessment of confounding factors. Therefore, while MIR9-3HG shows promise as a candidate biomarker, its clinical utility as a diagnostic or prognostic marker remains to be established in future studies.

## Data Availability

The datasets used and/or analyzed during the current study are available from the corresponding author on reasonable request.
